# Fungi as Turing automata with oracles

**DOI:** 10.1098/rsos.240768

**Published:** 2024-10-16

**Authors:** Andrew Schumann, Andrew Adamatzky, Jerzy Król, Eric Goles

**Affiliations:** ^1^ Department of Cognitive Science and Mathematical Modelling, University of Information Technology and Management in Rzeszow, Rzeszow, Poland; ^2^ University of the West of England, Unconventional Computing Laboratory, Bristol, UK; ^3^ University of Adolfo Ibáñez, Faculty of Engineering and Science, Santiago, Chile

**Keywords:** fungi, automata, Turing machine, oracle

## Abstract

In the article, we aim to understand the responses of living organisms, exemplified by mycelium, to external stimuli through the lens of a Turing machine with an oracle (oTM). To facilitate our exploration, we show that a variant of an oTM is a cellular automaton with an oracle, which aptly captures the intricate behaviours observed in organisms such as fungi, shedding light on their dynamic interactions with their environment. This interaction reveals forms of reflection that can be interpreted as a minimum volume of consciousness. Thus, in our study, we interpret consciousness as a mathematical phenomenon when an arithmetic function is arbitrarily modified. We call these modifications the hybridization of behaviour. oTMs are the mathematical language of this hybridization.

## 1. Introduction

We want to distinguish between the following two situations: (i) one is based on the existence of a biological substance that can be mechanically excited by external stimuli (this means this substance behaves like a deterministic automaton), and (ii) the other is the existence of a substance that reacts to external stimuli without determinism. This last case contains among others also the eventual conscious reactions of the substance to the stimuli. This does not mean that any conscious system cannot react algorithmically or mechanically, but rather that as long as its reactions are exclusively deterministic, we cannot distinguish them from those of (i). We want to understand the distinguished features of conscious behaviour along with the possibility to test the presence of a minimal level of consciousness involved in the reactions, with the opposition to unconscious reactions. The non-deterministic reactions as above can be also approached by the propagation of deformation waves through the biological substance. It is conceivable that the dosing of the substance with certain chemical agents (anaesthetics) can dampen the reactions, which would be an indirect fact of the presence of consciousness [1]. Even though such reactions are strong indications for consciousness being involved still they are neither sufficient for it nor explain the way of its functioning. That is why we want to understand the situation of the ‘minimal amount of consciousness’ contained in the bodies of such entities, based on purely computational means—through the test of non-determinism and the ability to hybridize one’s own behaviour.

To achieve our goals, we are developing a mathematical model that formally explains the situation of the presence of consciousness as a Turing machine with oracles (oTM) [2]. It is intentionally universal and extends Turing machines (TMs) [3] over higher uncomputable classes and can be applied in most situations where consciousness is present. We interpret consciousness as a general mathematical feature of various structures and does not necessarily imply any biological basis, so it may be applicable to artifical intelligence (AI) systems in the future. This separation from biology allows us to consider the ‘minimum volume of consciousness’, without referring to emotions or feelings, but also not excluding them.

One conclusion from the construction proposed is the possible algorithmic behaviour of entities or groups of entities, which do not automatically mean conscious behaviour. To see whether a reacting entity refers to its consciousness can be seen at the level of TMs or cellular automata. They both can interpret any algorithm but adding consciousness results in certain deformations of them. It follows from the presented theory that the precise deformation relies on taking oTMs or oracle cellular automata. Meanwhile, the formal theory of sets (ZFC) can give models for these machines with oracles [4]. We can show that such a formal construction develops many features usually attributed to conscious behaviour [5]. The core of the approach relies on the ability to interpret truly random stimuli internally as forcing extensions of models of ZFC (where oTMs or oracle cellular automata formulated in). The initial randomness is assimilated by the oracle which causes the growth (or shrinkage) of the entity along with its skills to understand and operate in the physical space [4,5]. This is precisely reflected by forcing the extension M[r] of a model M or taking the ground model N . This means that such a conscious entity operates uncomputably, extending the algorithmic capability of automata. Randomness is reversing into the extension of abilities and understanding.

Following [4,5], one can develop a direct analogy with conscious behaviour of complex organisms versus unconscious physical systems, S . Imagine two completely relative random physical stimuli $r_{1},r_{2}$ reacting on S . Their relative randomness, in addition to their individual random nature, also suggests that the stimuli would be completely independent as probabilistic events and would lead to independent outcomes (responses) of S . Consequently, when one of such stimuli enforces a response from S , it is still out of reach of S to draw any conclusion about the occurrence of other independent stimuli. The eventual response from the point of view of ‘what S can know without the inherent conscious viewing of the entire situation’ is still random and uncorrelated. This is because no inherent model of the external world is built in the case of unconscious reaction, or if a model is built, it is typically too weak to capture truly random external phenomena. However, when both random stimuli act on S in the presence of consciousness, the responses which arise can become logically dependent. Formally, this is an extended model of ZFC set theory, $M[r_{1}]$ (or $M[r_{2}]$ ), which currently houses $r_{2}$ ( $r_{1}$ ). From the point of view of oTM, such random stimuli become parts of the oracle, so thus the extended oracle acts as the source of the algorithmic process connecting $r_{1}$ and $r_{2}$ described now by the extended oTM. However, the Turing uncomputable class of oTM has to be eventually lifted as it is determined by the class of oracles, e.g. [6,7]. Thus, the presence (or absence) of TMs with oracles which can be uncomputably mortified in the process of responding to stimuli is a decisive for the presence of conscious reactions. Conscious organisms, when exposed to such random stimuli, interpret them in the internal model of the external world, where both stimuli are now represented as parts of the internal model. That is why we are focussed on the interpretability of formal oTM, along with the resulting higher classes of Turing uncomputabilities, when analysing conscious behaviour of living organisms.

There are many approaches to defining consciousness: philosophical, psychological, physiological, neurological, etc. In our work, hence, we rely on a mathematical approach that can be considered minimalistic, namely an approach from the point of view of robotics. A system is considered to have a minimal form of consciousness if it is capable of solving problems that do not have an algorithmic solution (from the point of view of computability theory, this can be reformulated as follows: the system solves unsolvable functions). For example, constructing Einstein’s theory of relativity is an algorithmically unsolvable problem. However, there are also plenty of everyday algorithmically unsolvable problems. For example, you find yourself in your friend’s kitchen for the first time and want to make coffee. Your task is to find the necessary ingredients for coffee in this kitchen and make it. This is a recognition problem. It can be somehow solved using deep learning, but it does not have an optimal algorithmic solution. Consciousness then is the ability to solve many problems for which there is no optimal algorithmic solution. Thus, most approaches to the study of consciousness follow a natural top-down way of thinking, in which human consciousness is defined by specifying its attributes, such as self-awareness and others. Here, we rather present a bottom-up approach to consciousness in general and focus on the ‘minimal’ content of consciousness, which is the case in very simple (compared with humans) organisms. Our goal is to formally understand this phenomenon so that this approach can (in principle) be applied to non-biological systems as well. In our previous work, we observed that oTMs defined in ZFC models satisfy general requirements for conscious behaviour, also specific to more advanced entities, such as learning, modelling of the external world or forms of self-awareness. Hence, an entity whose responses follow the action of oTMs satisfies the criterion of a ‘minimal’ conscious system in this extended sense.

To apply these findings to organisms like fungi would rely on:

- the functioning of some fungi can be interpreted by cellular automata and therefore by TMs (please see [8] and [9]). The question is whether they interpret the oTM or the oracle cellular automata and how to test it and under what conditions;
- we also need a proper definition of oracle cellular automata. It should be based on the implementation of true randomness (non-algorithmic) in cellular automata;
- the role of models of ZFC will be included since it is connected with non-algorithmic randomness (random forcing) [5]; and
- one should consider whether the modified cellular automata are (or are not) involved in the fungi’s activity when they react to stimuli and perform some algorithmic behaviour executed on uncomputable random data. This would depend on specific experimental data.

In §2, we provide some basic definitions of TMs and cellular automata to define a cellular automaton with an oracle. In §3, we define fungal cellular automata with oracles. In §4, we consider arithmetic functions and their codes in these oracle cellular automata of fungi. In §5, we analyse how to find the ‘minimum volume of consciousness’ in fungi.

## 2. Turing machines with oracles and cellular automata with oracles

**Definition 1**. *A TM can be defined as a mathematical construct represented by a 5-tuple* M=(Q,Σ,Γ,δ,F) *, where:*

—
$Q=\{q_{0},q,q^{\prime },\ldots \;\}$ *is a finite set of states, where* $q_{0}$ *is the start state; assume that* $q_{yes}$ *is to denote the accepting state from* Q *, and* $q_{no}$ *is to denote the rejecting state from* Q ;
—
Σ *is the input alphabet, for example* Σ={0,1} *; then we obtain 2-adic strings* $s\in \{0,1{\}}^{*}$ ;
—
Γ *denotes the tape alphabet, where* Σ *is a subset of* Γ *, indicating that the symbols in the input alphabet are also valid tape symbols. Additionally, the tape alphabet includes a special symbol* B *to represent a blank*;
—
δ:Q×Γ→Q×Γ×{L,R} *is a partial function defining the transition rules of the TM. For a given state* q *and tape symbol* s *,* $\delta (q,s)=(q^{\prime },t,\{L,R\})$ *either remains undefined or specifies a triple consisting of the new state* $q^{\prime }$ *, the symbol to write on the tape* t *, and the direction for the head to move, which can be either left (* L *) or right (* R *)*;
—
F⊆Q *consists of the accepting states.*

Let Σ={0,1} and $x\in \{0,1{\}}^{*}$ be a finite string regarded as an input. A computation on x begins with the input written on the tape. It is assumed that before and after this string there are contained only blank cells B . Following the definition provided, the TM initiates computation with its read/write head positioned at the leftmost non-blank cell, operating in the start state $q_{0}$ . The computation progresses by adhering to the transition rules denoted by δ , advancing step by step until (if at all) the head reaches an accepting state ( $q^{\prime }\in F$ ). Upon reaching an accepting state, should it occur, the sequence of symbols $y\in \Sigma^{*}$ inscribed on the tape is deemed the output of the computation, constituting a 2-adic number (natural number in a binary expansion).

Let each natural number x , i.e. the member of 𝐍 , be presented as a finite 2-adic string $x\in \{0,1{\}}^{*}$ , that is, $x=x_{0}x_{1}\ldots x_{n}=\sum_{i=0}^{n}x_{i}\cdot 2^{i}$ , where $x_{i}\in \{0,1\}$ . For example, $7=111=1+2+2^{2}$ . Assume that $\phi_{m}(x)$ denotes the computation of the TM, $M_{m}$ encoded by m such that the input tape is presented as a 2-adic string $x\in \{0,1{\}}^{*}$ . We write $\phi_{m}(x)=t$ if the computation of $M_{m}$ on x enters an answer $t\in \{0,1{\}}^{*}$ of the accepting states after applying finitely many transition rules. If there exists this t for $\phi_{m}(x)$ , then we write $\phi_{m}(x)↓$ . Meanwhile, if $\phi_{m}(x)$ never enters an accepting state, then we write $\phi_{m}(x)↑$ . Let $dom(\phi_{m})=\{x:\phi_{m}(x)↓\}$ . A function f:U→𝐍 , where U⊆𝐍 , is partially computable if there is a TM encoded by m such that $f(s)=t\Leftrightarrow \phi_{m}(x)=t$ , where the TM takes a string $s\in \{0,1{\}}^{*}$ as input, and halts with a string of $t\in \{0,1{\}}^{*}$ on the tape, and $\phi_{m}(x)↑\Leftrightarrow r\notin dom(f)$ . A function f:𝐍→𝐍 is computable if f is partially computable and total.

Let us introduce the characteristic function $\chi_{A}$ for a set A⊆𝐍 as follows:


$$\chi_{A}(x)::=\left\{ \begin{array}{ll} 1, & \text{if }x\in A\text{;}\\ 0, & \text{if }x\notin A\text{.} \end{array}\right.$$

The set A is computable if its characteristic function is computable and computably enumerable if it serves as the domain of a partial computable function. For instance, consider $K=\{x:\phi_{x}(x)↓\}$ . Then K is computably enumerable. However, K itself is not computable. To illustrate, we employ a diagonalization argument. Assuming K possesses a computable characteristic function, the function


$$f(x)::=\left\{ \begin{array}{ll} \phi_{x}(x)+1, & \text{if }x\in K\text{;}\\ 0, & \text{if }x\notin K\text{,} \end{array}\right.$$

must be computable. Nonetheless, for all x , it fundamentally differs from $\phi_{x}$ .

**Definition 2**. *An oracle machine is a TM* M *that has an access to an extra tape called the oracle tape or oracle that can solve the decision problem for an input* x *on the basis of oracle* $O\subseteq \{0,1{\}}^{*}$ *. This automaton is denoted by* $M^{O}$ *. We write the characteristic function of the set* O *on the tape:* $\ldots BBB\chi_{O}(0)\chi_{O}(1)\chi_{O}(2)\ldots$ *. The first function* $\chi_{O}(0)$ *is to say whether 0 belongs to* O *,* $\chi_{O}(1)$ *is to say whether 1 belongs to* O *, etc. Thus,* $\chi_{O}(0)\chi_{O}(1)\chi_{O}(2)\ldots$ *is an infinite 2-adic integer. The machine writes a query string* x *onto the query tape. Then the machine can calculate this string standardly, but also it can enter the query state* $q_{query}$ *to decide whether* x *is contained in the language* O *, and if so, it replaces everything on the query tape with the symbol 1 (this means that* $q_{query}$ *changes to the state* $q_{yes}$ *); otherwise, it replaces everything on the query tape with the symbol 0 (this means that* $q_{query}$ *changes to the state* $q_{no}$ *). After that, the oracle puts the machine into the answer state. This counts as one single step for the machine.*

Hence, whenever a TM, $M^{O}$ obtains x as an input, it may be informed whether x∈O to put $M^{O}$ in the ‘yes’ state (i.e. 1) if x∈O ; otherwise it puts $M^{O}$ in the ‘no’ state (i.e. 0) if x∉O .

**Definition 3**. *A cellular automaton is defined by a 4-tuple* A=(d,S,N,δ) , *where*:

—
d∈𝐍 *denotes the number of dimensions, and the elements of* ${𝐙}^{d}$ *are referred to as cells*;
—
S *is a finite set of elements representing the states of the automaton* A ; *the cells, which are members of* ${𝐙}^{d}$ , *take on values from* S ;
—
$N\subset {𝐙}^{d}\setminus \{0{\}}^{d}$ *is a finite ordered set containing* n=|N| *elements, known as the neighbourhood; it is assumed that each cell has an identical number of neighbours, which equals* n ;
—
$\delta :S^{n+1}\to S$ *is the local transition rule, where* n=|N| .

At each discrete time moment t=0,1,2,… , the arrangement of states across all cells is determined by the mapping $c_{t}:{𝐙}^{d}\to S$ , and the progression of the automaton unfolds through the sequence $c_{0},c_{1},c_{2},\ldots$ , which is defined as follows: $c_{t+1}(z)=\delta (c_{t}(z),c_{t}(\alpha_{1}),\ldots ,c_{t}(\alpha_{n}))$ , where $\left(\alpha_{1},\ldots ,\alpha_{n}\right)$ represents the neighbours of z . The initial configuration $c_{0}$ serves as the starting point and completely dictates the future behaviour of the automaton. This indicates that $c_{t+1}$ is entirely determined by $c_{t}$ . This property enables the construction of the function $G_{A}:C_{A}\to C_{A}$ , where $C_{A}$ denotes the set encompassing all potential configurations of the cellular automaton A (precisely, it represents all mappings from ${𝐙}^{d}$ to S , as each element of this set can function as the initial configuration $c_{0}$ , though not all elements may emerge in the evolution of other configurations). $G_{A}$ is termed the global function of the automaton A . We assert that a cellular automaton A computes the function f:X→Y if there exist injective mappings $g:X\to C_{A}$ and $h:Y\to C_{A}$ such that $G_{A}(g(x))=h(f(x))$ for each x∈X . It is noteworthy that $C_{A}$ (for |S|≥2 ) is not computably enumerable. We should recall that, according to the Church–Turing thesis, a function f defined on the natural numbers is computable if and only if it can be computed by a TM. However, there is an important assertion [10] that a cellular automaton, under certain conditions, is able to simulate a TM. This means that we can also analyse computable functions by means of cellular automata.

**Proposition 1**. *Let a TM* M *be presented by tape symbols* Γ={1,…,m} *and states* Q={1,…,n} . *We call these machines* (n,m) -*TMs. The transition function of* (n,m) *-TM is a function* δ:{1 , …, n}×{1,…,m}→{1,…,n}×{1,…,m}×{−1,0,1} . *Let the elements of* (n,m) *-TMs be given as follows*: $M=\underset{l\in 𝐙}{⋃}\prod_{k\in 𝐙}D_{k}^{l}$ , *where* $D_{k}^{l}=𝐍$ for l≠k *and* $D_{k}^{k}=(𝐍\times 𝐍)$ . *An element* $\left(\ldots ,i_{l-1},(i,j),i_{l+1},\ldots \;\right)$ *from* $\prod_{k\in 𝐙}D_{k}^{l}$ *shows that a TM has a state* j *and reads a symbol* i *at a position* l . *The content of the tape of the TM is given by* $\left(\ldots ,i_{l-1},i,i_{l+1},\ldots \;\right)$ . *Assume that* 𝐍 *is the set of states of one-dimensional cellular automata of radii one* (CA). *Then the set* ${𝐍}^{𝐙}$ *contains every configuration of CA*. *Let the elements of* M *are mapped on* ${𝐍}^{𝐙}$ *by a function* F . *It is locally defined by two functions* $g:𝐍\to {𝐍}^{p}$ *and* $h:({𝐍}^{2})\to {𝐍}^{q}$ *as follows*:


$$F((\ldots ,d_{l-1},(i,j),d_{l+1},\ldots \;){)}_{pk+s}=g(d_{k}{)}_{s}$$

*for* s=1,...,p *and* k<l ,


$$F((\ldots ,d_{l-1},(i,j),d_{l+1},\ldots \;){)}_{pl+s}=h(d_{l}{)}_{s}$$

*for* s=1,…,q *and*


$$F((\ldots ,d_{l-1},(i,j),d_{l+1},\ldots \;){)}_{p(k-1)+q+s}=h(d_{k}{)}_{s}$$

*for* s=1,...,p *and* k>l .

*Let* D *be a cellular automaton. Then* D *simulates* M *with order* (p,q) *and delay* t *if there is* F *such that for any* x∈M *, there exists* $t^{\prime }\leq t$ *such that* $G^{t^{\prime }}(F(x))=F(T(x))$ *, where* G *is the global transition function of the cellular automaton* D *and* T *is the partial function defined on* M *that represents* M .

*Proof*. See [10].∎

Let us assume that a function f:X→Y is computed by a TM. It means that on the input x∈X it should give the output y=f(x)∈Y . However, a TM with an oracle can give an answer without calculation based on what is contained in the set O , namely, whether f(x) is in this set. Let f(x)∈O and there exist injective mappings $g:X\to C_{A}$ and $h:Y\to C_{A}$ for a cellular automaton A such that $G_{A}(g(x))=h(f(x))$ for each x∈X . Then we say that A has the oracle f(x) .

**Definition 4**. *An oracle cellular automaton is a 5-tuple* $A^{O}=(d,S,N,\delta ,O)$ , *where* d,S,N,δ *are the same as in definition 3 and* O⊆ ∗S *is a subset of all infinite strings (i.e. the strings of the infinite length) built on* S . Let S={0,1} . *Then the oracle* O *consists of some infinite 2-adic strings from the set* ${𝐙}_{2}$ *of all 2-adic integers* (*meanwhile, the set* ${𝐙}_{2}$ *is infinite* ^1^). *Each member* o∈O *is interpreted as an additional transition rule, different from* δ *and consisting of an infinite number of additional transition rules* $\delta_{t}$ , *applied at the time step* t=0,1,2,…,∞ , *namely*:


$$o::=(\delta_{0},\delta_{1},\delta_{2},\ldots )=\delta_{0}\delta_{1}\delta_{2}\ldots$$

*Let us note that for* S={0,1} *,* $o\in {𝐙}_{2}$ *.*

*We will distinguish between two types of these members of* O *: (i) standard functions denoted by* $\;*\delta_{0}$ *, for which* $\delta_{0}=\delta_{1}=\delta_{2}=\ldots$ *in* $\left(\delta_{0},\delta_{1},\delta_{2},\ldots \;\right)$ *, where* = *is an equality of two functions* $\delta_{i}$ *and* $\delta_{j}$ *for* i≠j *, which are equal if they have the same domain and the same codomain, and if for every* x *in the domain,* $\delta_{i}(x)=\delta_{j}(x)$ *; (ii) and non-standard functions denoted by* $\left[\delta^{\prime }\right]$ *, for which there exist* i,j∈𝐍 *such that* $\delta_{i}^{\prime }\neq \delta_{j}^{\prime }$ *in* $\left(\delta_{0}^{\prime },\delta_{1}^{\prime },\delta_{2}^{\prime },\ldots \;\right)$ *. The difference between* $\;*\delta_{0}$ *and* $\left[\delta^{\prime }\right]$ *is as follows. If* $o=\;*\delta_{0}$ *, this means that at each time step* t *there is applied the same transition rule* $\delta_{0}$ *. If* $o=[\delta^{\prime }]$ *, this means that at each time step* t *there is applied the different transition rule* $\delta_{t}^{\prime }$ *from the infinite string* $\left[\delta^{\prime }\right]$ *. In other words, if we apply* $o=[\delta^{\prime }]$ *, then we can change the transition rule at each time step* t *.*

*Let* $c_{t}:{𝐙}^{d}\to S$ *for* t=0,1,2,… *, then the evolution of the automaton* $A^{O}$ *in accordance with the member* o∈O *means a sequence* $c_{0}$ *,* $c_{1}$ *,* $c_{2}$ *, … defined as follows:* $c_{t+1}(z)=\delta_{t}(c_{t}(z),c_{t}(\alpha_{1})$ *, …,* $c_{t}(\alpha_{n}))$ *, where* $\left(\alpha_{1},\ldots ,\alpha_{n}\right)$ *are the neighbours of* z *and* $\delta_{t}$ *is taken at the time step* t *from* $\left(\delta_{0},\delta_{1},\delta_{2},\ldots \delta_{t},\ldots \;\right)$ *.*

*Let* $x_{t}$ *be an input of the automaton* $A^{O}$ *at the time step* t=0,1,2,… *. It can be calculated either in the standard way, by applying* δ *of* A *, or by invoking the oracle member* o∈O *, that is, by applying either* $o=\;\;*\delta_{0}$ *or* $o=[\delta^{\prime }]$ *. In other words, if* $\delta_{t}(x_{t})$ *is defined for* $\delta_{t}$ *from* o *, then the evolution of the automaton* $A^{O}$ *takes place in accordance with the oracle* $(\delta_{t},\delta_{t+1},\ldots \;)\in O$ *. If not, then* $A^{O}$ *behaves like* A *without* O *.*

It should be noted that if the function is non-standard (i.e. it is $\left[\delta^{\prime }\right]$ ) and the automaton follows it, then this means that the evolution of such an automaton changes the transitive rule at some or all steps. This allows us to simulate uncomputable functions with cellular automata. We can amplify this as follows.

**Definition 5**. *Let* $A^{O}=(d,S,N,\delta ,O)$ *be an oracle cellular automaton, where* S *is a ring (e.g.* S=𝐙 *or* S=𝐐 *). Then, its member* o∈O *is called a hybridization of* A *if and only if*


$$o::=(\delta_{0}(x_{0})+\epsilon_{0},\delta_{1}(x_{1})+\epsilon_{1},\delta_{2}(x_{2})+\epsilon_{2},\ldots \;)$$

*for the inputs* $x_{t}$ *at the time step* t=0,1,2,… *, where* $\epsilon_{t}<|\delta_{t}(x_{t})|$ *for all* t=0,1,2,…

If the oracle function is standard (i.e. it is $\;*\delta_{0}$ ) and the automaton follows it, then it behaves standardly (without changing its transition rules), but only until the moment when there will be another appeal to the oracle. If, as a result of this new request, the automaton begins to follow the new function, then the transitive rule of the automaton will change.

The main idea behind the oracle cellular automata is that these automata have ways to change the transitive rule over time (definition 4). This is not possible with conventional cellular automata. An extreme case of such permanent change is automaton hybridization (definition 5). In computer science, such cellular automata with an oracle have not been used so far. However, they open up wide possibilities for simulating biological systems. The fact is that biological systems such as fungi exhibit a remarkable duality in their behaviour, demonstrating characteristics of both rigidity^2^ and adaptability. On the one hand, fungi can embody some automata in their behaviour [8,9]. This means they can operate in a highly predictable manner, responding to specific stimuli with predefined, programmed responses. Such behaviour is akin to mechanical or computational systems, where input consistently produces a specific output, reflecting the deterministic nature of automata. However, on the other hand, biological systems and even one-cellular organisms [11] can also change their behaviour for the same stimuli. Unlike simple automata, biological entities possess the ability to learn from their experiences, adapt to new environments and modify their responses based on internal and external factors. This adaptive behaviour showcases the dynamic nature of biological systems,^3^ enabling them to cope with changing environments, optimize their survival strategies and exhibit what can be perceived as a form of decision-making or consciousness. Hence, while biological systems can display the mechanical predictability of automata, they are also capable of remarkable flexibility and adaptability, altering their behaviour in response to the same stimuli under different circumstances. This dual capability highlights the sophisticated nature of biological systems and underscores the applicability of cellular automata with an oracle not only in simulating the adaptability of fungi and other creatures, but also in implementing biologically inspired mechanisms of decision-making in AI.

## 3. Fungal cellular automata with oracles

Every living organism can be represented as an automaton, in which the inputs are stimuli (such as chemotaxis) and the outputs are motor reactions [12]. Meanwhile, we can observe a wide diversity in the chemotactic behaviour of even one-cellular organisms [13]. Mathematically, this can be explained by the fact that a living organism, although presented in the form of an automaton, is not deterministic. Under certain conditions, the number of outputs in such an automaton exceeds the number of inputs [14]. This can be interpreted as a feature of living nature—even one cell is able to respond to the environment with greater variability than the set of perceived signals itself. At best, a deterministic automaton approximates real behaviour with a certain error. The problem is that a fungus (mould, etc.) can implement some algorithms, but not all (e.g. some logic gates), only by a certain percentage—e.g. from 70% to 90%. In some cases, they have no algorithmic solution at all. Then it is worth specifying an oTM (or a cellular automaton with an oracle), where this oracle will give out random numbers, in the range of which the numbers from the algorithms will be located. For example, let the TM generate a logic gate with a probability of 75% for fungi, then an appropriate oTM may generate random numbers, among which there is this logic gate. This means that the TM with the oracle is now implemented experimentally by 100%.

Fungi use a sophisticated communication and information processing system, notably through their mycelium networks [15]. Filamentous fungi such as *Aspergillus*, *Penicillium*, *Fusarium*, *Verticillium* and *Phanerochaete* (see [16]), and other species of Basidiomycota and Zygomycota grow by extending hyphae at their tips.

Filamentous fungi within the Ascomycota phylum possess porous septa that facilitate cytoplasmic streaming [17,18]. Following hyphal injury, Woronin bodies serve to occlude these septal pores, preventing excessive cytoplasmic leakage [19–23]. Initially, Woronin bodies tend to be localized at the apex when they first form [24–26]. Subsequently, these bodies undergo transportation and anchoring to either the cell cortex (*Neurospora crassa*, *Sordaria fimicola*) or in close proximity to the septum (*Aspergillus oryzae*, *Aspergillus nidulans*, *Aspergillus fumigatus*, *Magnaporthe grisea*, *Fusarium oxysporum*, *Zymoseptoria tritici*) until they are translocated to the septal pore, driven by cytoplasmic flow or ATP depletion [22–25,27–31]. Those Woronin bodies that are not anchored at the cellular cortex or the septum remain in the cytoplasm and exhibit high mobility (*A. fumigatus*, *A. nidulans*, *Z. tritici*) [24,26,28]. The occlusion of septal pores can be triggered by bulk cytoplasmic flow [28] or various developmental and environmental cues, such as cell wall puncturing, high temperature, carbon and nitrogen starvation, high osmolarity and low pH [32].

Additionally, intact hyphae can also have their septa blocked by Woronin bodies. Septal pore occlusion may be prompted by factors such as the growth process and stress conditions (high temperature, as well as carbon and nitrogen starvation; see [32–34]). Thus, the state of the pores (their openness or closure) is a response to external stimuli, which can be favourable (attractants) or stressful (repellents). Under conditions of attractants, the pores are open, and under conditions of repellents, the pores are closed. Each such external stimulus changes the configuration of the pores, and hence the computational architecture itself. We will further show that each such external stimulus that changes the computation can be understood as an oracle.

**Figure 1.**
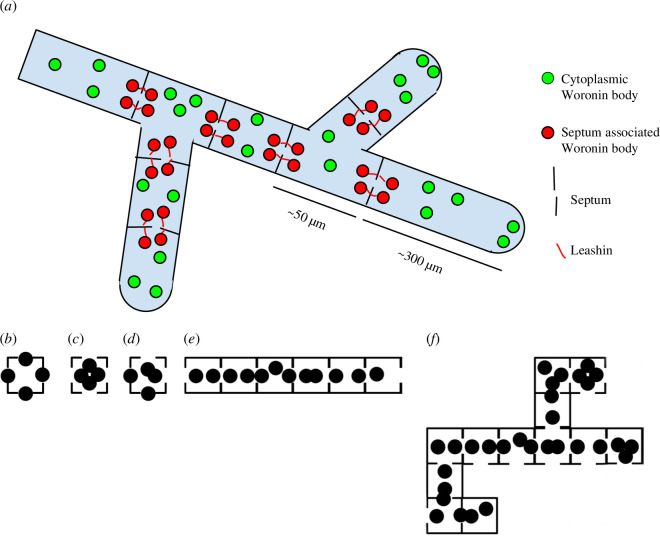
Fungal cellular automata. (*a*) Septate hypha with pores. (*b*) Cell of the hypha, in which all pores are closed. (*c*) All pores of the cell are open. (*d*) Only south and west pores of the cell are open. (*e*) One-dimensional fungal cellular automaton. (*f*) Two-dimensional fungal cellular automaton.

A depiction of the mycelium featuring Woronin bodies is illustrated in figure 1. It is a septate structure, in which pores can be either open or closed. The compartments within the mycelium have a different number of adjacent compartments, forming the elementary units of fungal cellular automata (figure 1*b−d*)—one cell that has potentially four pores on four different sides and these pores can be either open or closed. We assume that these compartments can be assembled into one-dimensional structures (figure 1*e*) or two-dimensional (figure 1*f*). Thus, in our study, we investigate a cellular automaton operating in a d -dimensional grid ${𝐙}^{d}$ with some neighbourhoods, characterized by a set of states S and a global function G . Each cell in the grid possesses four sides that can be open or closed. Open sides facilitate information exchange between adjacent cells, while closed sides imply mutual ignorance. By controlling the openness of sides, the dynamic behaviour of the automaton varies. We consider specific configurations where vertical and horizontal sides of the grid may be initially open or closed, and in subsequent steps, this situation may be different. An external stimulus is what changes the openness and closeness of the pores on the corresponding side. We assume that only one stimulus can be encountered on each side for all automaton cells, and it can be both an attractant and a repellent. As a result, eight stimuli are possible: $A_{\;n}$ —attractant to the north of the automaton; $A_{\;s}$ —attractant to the south of the automaton; $A_{\;w}$ —attractant to the west of the automaton; $A_{\;e}$ —attractant to the east of the automaton; $R_{\;n}$ —repellent to the north of the automaton; $R_{\;s}$ —repellent to the south of the automaton; $R_{\;w}$ —repellent to the west of the automaton; $R_{\;e}$ —repellent to the east of the automaton.

This research focuses on particle flows, where each cell of the automaton contains a finite number of particles or chips distributed according to certain rules. The evolution occurs synchronously, with each site simultaneously losing and gaining chips. This mechanism may be simulated by cellular automata [8,9]. In this context, the state set includes S={0,1,2,…}⊂𝐍 representing the number of particles. However, at the same time, we suppose that all sites (cells) cannot have more than p−1 chips. As a result, we get p -adic strings, in which the ciphers in the string can change at each calculation step.

Let us fix p∈{2,3,5} for a fungal cellular automaton. If a site $v\in {𝐙}^{2}$ holds $x_{v}=p-1$ chips, the following actions occur, when all of the four pores of v are open:


(3.1) $$\begin{array}{rl} & x_{v}^{\prime }=x_{v}-(p-1)\\ & \exists u\in N_{v}\Rightarrow x_{u}^{\prime }=x_{u}+1. \end{array}$$

Here, $N_{v}$ denotes the von Neumann neighbourhood of site v , and $x_{v}^{\prime }$ reflects the updated value of $x_{v}$ . However, if we have four pores open, but we are dealing with 2-adic strings, then it is not clear at which pore from four a chip can leak. This makes the machine non-deterministic. However, let us consider only deterministic automata. Namely, let us assume that the number of pores for all cells of the automaton is no more than p−1 . For example, for 2-adic strings in all cells only one pore is open.

By incorporating the notion of open or closed sides proposed here, the rule for p adjusts as follows:


(3.2) $$\begin{array}{rl} & x_{v}\geq p-1\Rightarrow x_{v}^{\prime }=x_{v}-\alpha \\ & \forall u\in N_{v}\text{ such that the gate is open}\\ & \Rightarrow x_{u}^{\prime }=x_{u}+1, \end{array}$$

where α≤p−1 denotes the number of open gates.

When the rule is simultaneously applied to every site, the new state at a site v is determined by:


$$x_{v}^{\prime }=x_{v}-\alpha +\beta .$$

Here, β represents the chips received by site v from its open and firing neighbours, the number of which does not exceed p−1 .

**Figure 2.**
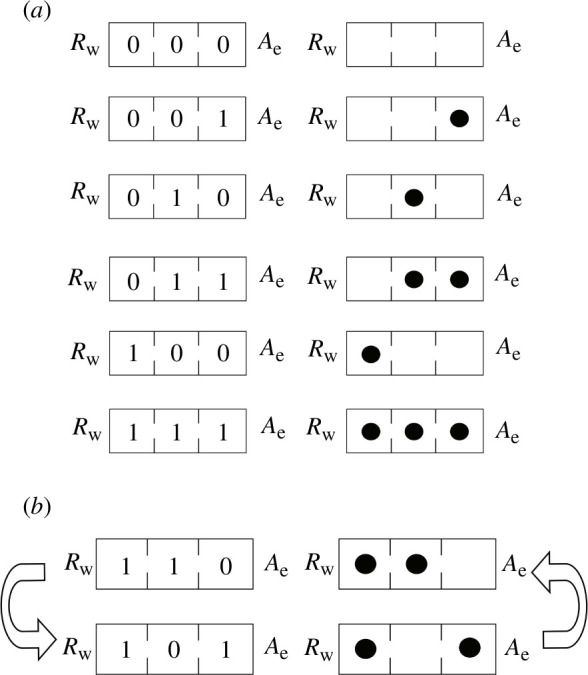
Fungal cellular automaton for the Fredkin gate. The pore of the leftmost cell is blocked owing to the repellent $R_{\;w}$ . (*a*) Six 2-adic strings which do not change. (*b*) Two 2-adic strings which form an eternal cycle: [1,1,0] is mapped to [1,0,1] and [1,0,1] is mapped to [1,1,0] .

Let us consider an example of a cellular automaton consisting of one-dimensio­nal horizontal 2-adic strings three cells long: $\left[x_{1},x_{2},x_{3}\right]$ , where $x_{1},x_{2},x_{3}\in \{0,1\}$ . Since they are horizontal, external stimuli for them can only be from the right and left (from the west and from the east). Let us assume that at the moment there is a repellent on the left ( $R_{\;w}$ ) and an attractant on the right ( $A_{\;e}$ ). Let us also suppose that chips can only move if there are at least two of them within the framework of one string. Then the fungal cellular automaton at this moment (namely with $R_{\;w}$ and $A_{\;e}$ ) simulates the Fredkin gate. Recall that this is a reversible logic gate, which for the input $[x_{1},x_{2},x_{3}]\in \{0,1{\}}^{3}$ gives the output $[x_{1}^{\prime },x_{2}^{\prime },x_{3}^{\prime }]\in \{0,1{\}}^{3}$ in accordance with table 1. It is the following rule: $x_{1}^{\prime }=x_{1}$ , $x_{2}^{\prime }$ = $\mathrm{OR}(\mathrm{AND}(\mathrm{NOT}x_{1}$ , $x_{2})$ , $\mathrm{AND}(x_{1}$ , $x_{3}))$ , and $x_{3}^{\prime }$ = $\mathrm{OR}(\mathrm{AND}(x_{1}$ , $x_{2})$ , $\mathrm{AND}(\mathrm{NOT}x_{1}$ , $x_{3}))$ , i.e. we deal with the three inputs $x_{1}$ , $x_{2}$ , $x_{3}$ , and the three outputs $x_{1}^{\prime }$ , $x_{2}^{\prime }$ , $x_{3}^{\prime }$ . The fungal cellular automaton maps [0,0,0] to [0,0,0] , [0,0,1] to [0,0,1] , [0,1,0] to [0,1,0] , [1,0,0] to [1,0,0] , because all these strings contain only one unit. The 2-adic string [0,1,1] includes two units, but it can be mapped only to itself [0,1,1] , since there is the repellent $R_{\;w}$ on the left side of the string and its first site cannot receive the unit from its right neighbour. The 2-adic string [1,1,1] cannot also change. It is complete. Meanwhile, the 2-adic string [1,1,0] is mapped to [1,0,1] and the string [1,0,1] is mapped to [1,1,0] (see figure 2).

**Table 1.** The Fredkin gate in the permutation matrix form. (The input [000] is mapped to the output [000], [001] is mapped to [001], etc.)

	000	001	010	011	100	101	110	111
000	1	0	0	0	0	0	0	0
001	0	1	0	0	0	0	0	0
010	0	0	1	0	0	0	0	0
011	0	0	0	1	0	0	0	0
100	0	0	0	0	1	0	0	0
101	0	0	0	0	0	0	1	0
110	0	0	0	0	0	1	0	0
111	0	0	0	0	0	0	0	1

Let us remember that the Fredkin gate is considered universal [35], which means that any logical or arithmetic operation can be constructed entirely of Fredkin gates. This universality signifies that Fredkin gates can be used to build any possible computation, making them a powerful tool in the realm of computing. The fact that fungal mycelia can embody Fredkin gates suggests that such mycelia can implement various logical and arithmetic functions that can be expressed through a series of Fredkin gates. However, mycelium has its own physical limitations for implementing, e.g. a fairly long sequence of Fredkin gates. For example, it may require too high a density of fungi to support a given computational scheme, or too large a mycelium. However, if we ignore any physical limitations of the mycelium, owing to its organization as a special cellular automaton, it is able to implement any arithmetic function.

Summing up, the fungal mycelium as an abstract automaton can implement all logical and arithmetic functions, since it implements the Fredkin gate. However, this does not mean that we can observe all these possible implementations of TMs (i.e. logical and arithmetic functions) in reality. It has been established [36] that biological organisms usually implement only some logical functions as their natural response. However, through approximation and enhancement of the artificial environment, biological organisms can be made to perform various logical and arithmetic operations.

Based on this example, we see that cellular automata have external inputs (repellents and attractants of the mycelium) and internal inputs ( p -adic strings, which are transformed owing to the presence or absence of pores in the cells). Now let us define these automata in the general case.

**Definition 6**. A fungal cellular automaton with an oracle is a 5-tuple $A_{F}^{O}=(d,S,N,\delta ,O)$ , where

—
d∈𝐍 *is a number of dimensions; e.g. if* d=1 *, then the automaton is one-dimensional*;
—
S={0,1,…,p−1} *is a finite set of elements called states. Each state shows a number of chips in an appropriate cell. We also assume that each cell has no more than* p−1 *open pores and the state* p−1 *of* $v\in {𝐙}^{d}$ *means that this cell* v *is active and its chips may leak through pores, otherwise* v *is inactive*;
—
$N\subset {𝐙}^{d}\setminus \{0{\}}^{d}$ *is a finite ordered set consisting of* n=|N|≤p−1 *elements,* N *is said to be a neighbourhood; but in the fungal cellular automaton we see that* n *is changeable for some cells at some time moment* t=0,1,… *and depends on the number of open pores for such a cell*;
—
δ *:* $S^{n+1}\to S$ *is the local transition rule. Let* $N_{v}$ *be a neighbourhood of the cell* v *, such that* $|N_{v}|=n$ *. We assume that the pores to the cells of* $N_{v}$ *from* v *can be open. Let* α *be a number of open pores to* $N_{v}$ *. Then* δ *maps the state* $x_{t}(v)$ *of* v *to the new one* $x_{t+1}(v)$ *as follows:*

$x_{t+1}(v)=\left\{ \begin{array}{ll} x_{t}(v)-\alpha +\beta , & \text{if }x_{t}(v)=p-1\geq \alpha \text{ and }\alpha \text{ pores are open }\\ & \text{and }\beta \leq \alpha \text{ neighbour cells are active;}\\ x_{t}(v)+\beta , & \text{if }x_{t}(v)<\alpha \text{ and }\alpha \text{ pores are open }\\ & \text{and }\beta \leq \alpha \text{ neighbour cells are active;}\\ x_{t}(v), & \text{if pores are closed;}\\ x_{t}(v), & \text{if }x_{t}(v)<\alpha \text{ and }\alpha \text{ pores are open,}\\ & \text{but neighbour cells are inactive.} \end{array}\right.$

*If after calculations, according to this* δ *, the new state* $x_{t+1}(v)=x>p-1$ *, then we take* $x_{t+1}(v)=(p-1)$ ;
—
O *is the oracle consisting of external stimuli:*

$$o::=(St_{0},St_{1},St_{2},\ldots ),$$

*where each* $St_{i}$ *consists of repellents and attractants that make the pores of cells open and closed, which changes the computing architecture of the automaton, since it changes the number of* n *elements in the neighbourhood* $N_{v}$ *of the cells* v *. Suppose a request to the oracle at time* t *means that the automaton is checking to see if it can follow* o *.*

Changing the architecture of a fungal cellular automaton in response to stimuli can be interpreted as the system’s self-positioning in its environment, which is inherently unpredictable. Stimuli appear randomly, but they cause changes within the system that are subsequently integrated into its functioning. Some pores open, some close and the machine produces other p -adic strings.

## 4. Arithmetic functions and their codes in fungal cellular automata with oracles

Let us examine discrete time, denoted by t=0,1,2,… , where at each time step t , the cellular automaton $A_{F}^{O}$ has no more than n inputs (active cells) as a reaction to external stimuli and no more than p−1 particles or chips in each cell. Then the fungal reactions may be analysed as an arithmetic function $f_{p^{n}}(x)=y$ , where $x,y\in \{0,1,\ldots ,p^{n}-1\}$ . For instance, in the Fredkin gate (depicted in figure 2), we deal with the arithmetic function $f_{2^{3}}$ , where the inputs and outputs from table 1 are rewritten as natural numbers: $x_{0}x_{1}x_{2}=\sum_{i=0}^{2}x_{i}\cdot 2^{i}$ , which is a binary expansion. For instance, 000=0 and $111=\sum_{i=0}^{2}1\cdot 2^{i}=7$ . Therefore, if we have n inputs at time step t and p -adic strings, then $A_{F}^{O}$ calculates an arithmetic function $f_{p^{n}}$ at this t . As a consequence, if the fungus is exposed to n stimuli or less at each time step t=0,1,2,… , we derive a sequence of functions:


$$f_{p^{n}}^{t=0},f_{p^{n}}^{t=1},f_{p^{n}}^{t=2},\ldots ,$$

where at each time step t=i , the arithmetic function $f_{p^{n}}^{t=i}$ may differ. This sequence is an oracle o of $A_{F}^{O}$ which can be interpreted as a p -adic valued function:


(4.1) o::=f(α)=β,

where $\alpha =\alpha_{0}\alpha_{1}\alpha_{2}\ldots$ and $\beta =\beta_{0}\beta_{1}\beta_{2}\ldots$ , such that $f_{p^{n}}^{t=i}(\alpha_{i})=\beta_{i}$ for each i=0,1,2,… . The quantities α and β are expressed in $p^{n}$ -adic form, with $\alpha =\sum_{i=0}^{\infty }\alpha_{i}\cdot (p^{n}{)}^{i}$ and $\beta =\sum_{i=0}^{\infty }\beta_{i}\cdot (p^{n}{)}^{i}$ , where $\alpha_{i},\beta_{i}$ run over the set $\left\{0,\ldots ,p^{n}-1\right\}$ for each i=0,1,2,…

We can list all the arithmetic functions that can be executed within fungal networks, formalized as $A_{F}^{O}$ , by following a structured approach. To begin, we partition all arithmetic functions over ${𝐙}_{p}$ for varying values of p . For each p , there exists a distinct enumeration of all arithmetic functions corresponding to a specific time step t . Each function $f_{p^{n}}^{t}$ signifies one of the potential permutations of the numbers $0,1,\ldots ,p^{n}-1$ , and it can be uniquely distinguished by a designated code:


(4.2) $$\lceil f_{p^{n}}^{t}\rceil =\sum_{i=0}^{t}\left(\sum_{j=1}^{p^{n}-1}c_{ji}\cdot j!\right)\cdot ((p^{n}-1)!{)}^{i}.$$

In this context, $c_{ji}$ represents the tally of instances in the i th permutation where the value j appears to the right of another value smaller than j . For example, in the permutation of the Fredkin gate (0,1,2,3,4,6,5,7) , we observe:


$$\sum_{j=1}^{2^{3}-1}c_{j}\cdot j!=0\cdot 1!+0\cdot 2!+0\cdot 3!+0\cdot 4!+0\cdot 5!+1\cdot 6!+0\cdot 7!=720.$$

Supposing the Fredkin gate has been used twice with t=1 , we obtain:


$$\sum_{i=0}^{1}\left(\sum_{j=1}^{2^{3}-1}c_{ji}\cdot j!\right)\cdot ((2^{3}-1)!{)}^{i}=720+720\cdot 7!=3629520.$$

For the function $f_{p^{n}}^{\infty }$ , its code is determined by:


(4.3) $$\begin{array}{ccc} & \lceil f_{p^{n}}^{\infty }\rceil =\sum_{t=0}^{\infty }\left(\sum_{j=1}^{p^{n}-1}c_{jt}\cdot j!\right)\cdot ((p^{n}-1)!{)}^{t}. & \end{array}$$

Therefore, each p -adic valued arithmetic function $f_{p^{n}}$ as described in equation (4.1) and coded by equation (4.3) symbolizes an infinite path followed by a fungus in its reactions to the environment (about p -adic valued logic please see [37]). This path is subject to the condition of encountering no more than n inputs at each time step t with no more than p−1 particles or chips in each cell.

It is worth noting that the set O of the fungal cellular automaton $A_{F}^{O}$ can contain arithmetic functions $f_{p^{n}}$ along with their codes $\left\lceil f_{p^{n}}^{t}\right\rceil$ as an oracle.

## 5. Diagonalization argument in fungal cellular automata with oracles

According to proposition 1, the term of computability or decidability of a set X can be formulated in the context of cellular automata A (see definition 3), which exhibit the following behaviour:


$$A(x)=\left\{ \begin{array}{ll} 1, & \text{if }x\in X\text{;}\\ 0, & \text{otherwise.} \end{array}\right.$$

Let $F_{p}^{t}$ contain arithmetic functions $f_{p^{n}}^{t}$ . We postulate that this $F_{p}^{t}$ is decidable, indicating the existence of a cellular automaton $A_{i}$ that determines whether a function $f_{p^{n}}^{t}$ belongs to $F_{p}^{t}$ given its code $i=\lceil f_{p^{n}}^{t}\rceil$ . In essence, this implies the existence of a cellular automaton A capable of processing an input $\left\langle i,x\rangle =\langle \lceil f_{p^{n}}^{t}\rceil ,f_{p^{n}}^{t}\right\rangle$ and producing $A_{i}(x)$ as output.

The collection $K=\{\langle i,x\rangle :A_{i}(x)$ terminates} is defined as a halting set. If the computation concludes, then ⟨i,x⟩ belongs to K .

**Proposition 2**. *The set* K *is not decidable.*

*Proof.* Using diagonalization argument, we can demonstrate this. Assuming K is decidable, let $A_{0}$ be a cellular automaton that decides K :


$$A_{0}(x)=\left\{ \begin{array}{ll} 1, & A_{i}\text{ halts;}\\ 0, & \text{otherwise.} \end{array}\right.$$

Given that $A_{0}$ is a cellular automaton with a specific code e , we have $A_{\;e}=A_{0}$ . Let’s introduce $F_{p}^{\prime t}=\{i:A_{i}(i)\neq 1\}$ , a collection that is undecidable for any given i . Consequently, the set K , which includes all pairs ⟨i,i⟩ where $A_{i}(i)\neq 1$ , is undecidable as well.∎

However, O of a fungal cellular automaton $A_{F}^{O}$ can contain both arithmetic functions $f_{p^{n}}^{t}$ and their codes $\left\lceil f_{p^{n}}^{t}\right\rceil$ , as we said. It follows from this that the oracle O is undecidable. TMs (cellular automata) with an oracle O calculate based on the uncomputable. This is a good analogy with consciousness as a mechanism of reflection and self-reflection, which cannot be described within the framework of a domain-specific language, i.e. within the framework of a language for describing reality. Hence, the diagonalization argument shows that based on arithmetic functions and their codes, we cannot in general answer the question of whether a given function is solvable. This mathematical fact of proposition 2 can be interpreted in terms of consciousness. Even if we take arithmetic functions, which by definition are decidable, our reflection on their decidability is not decidable. Reflection regarding the computable is no longer computable.

This phenomenon leads to a scenario where the number of outputs surpasses the number of inputs, termed as ‘hybrid action’. Such reactions, characterized by having n inputs and m≥n outputs, may constitute a ‘hybrid action’, which, by definition, is unsolvable if m>n and falls within the scope of proposition 2. An illustrative example is as follows:


(5.1) $$A(d(f_{p^{n}}^{t}))=\left\{ \begin{array}{ll} 1, & \text{if }d(f_{p^{n}}^{t})\geq f_{p^{n}}^{t}\text{ for all }f_{p^{n}}^{t}\text{ at }t\text{ with }n\text{ inputs;}\\ 0, & \text{otherwise.} \end{array}\right.$$

This $d(f_{p^{n}}^{t})$ is called a weak diagonalization of $f_{p^{n}}^{t}$ . If $d(f_{p^{n}}^{t})>f_{p^{n}}^{t}$ for all $f_{p^{n}}^{t}$ at t with n inputs in equation (5.1), then this diagonalization is called strong.

The statement of proposition 2 concerns the uncomputability of the set O of $A_{F}^{O}$ in the general case, but it is important for us to be able to model finite sets of natural numbers. For example, when modelling mycelium, we are dealing with very small sets, and it is important for us to be able to see hybridization on them (the minimum level of reflection and self-reflection). Equation (5.1) allows us to do this easily.

Each separate horizontal or vertical string of fungal automata can be regarded as a p -adic number, where p−1 is a maximal number of chips in all cells (see definition 6). Let ∘ denote a composition of two different strings and suppose that this operation is associative and commutative. So, if a,b are two vertical or horizontal p -adic strings, possibly of different lengths, then a∘b is a result of their composition. Assume that there exists an identity element e such that for all a we have a∘e=a . This e is presented by any p -adic string in which all the cells contain only 0 and their pores are closed, i.e. these cells are inactive. Also, we have a strong diagonalization $\bar{e}$ such that for all a we have $a\circ \bar{e}=\bar{e}$ . This $\bar{e}$ is presented by any p -adic string in which all the cells contain only p−1 , indicating that all cells are active. Let a be a function $f_{p^{n}}^{t}$ . Then $\bar{e}$ is $d(f_{p^{n}}^{t})$ for it from equation (5.1) and furthermore $\bar{e}>a$ for all a , therefore the diagonalization $\bar{e}$ is termed as strong.

**Figure 3.**
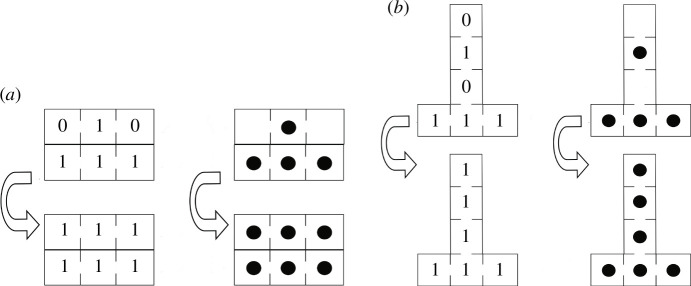
Composition $a\circ \bar{e}=\bar{e}$ for a=[0,1,0] . (*a*) a is horizontal. (*b*) a is vertical.

Let us exemplify the composition $a\circ \bar{e}=\bar{e}$ for a=[0,1,0] . Then $\bar{e}$ is 2-adic. This a can be vertical or horizontal, but in any case its composition with $\bar{e}$ gives $\bar{e}$ (see figure 3). It is worth noting that $\bar{e}$ is greater than any finite 2-adic string consisting of only 1.

We can note that sometimes the composition ∘ behaves like addition for some p -adic strings. Suppose that a=[0,1,0] and b=[1,0,1] and both are horizontal. Then a∘b=a+b=[1,1,1] . However in general, composition does not give addition. In this structure, we do not have inverse operation (subtraction for addition), since in $A_{F}^{O}$ we are dealing with chips in cells or their absence and we do not have negative values. Thus, the composition ∘ forms an Abelian monoid with a diagonalization $\bar{e}$ . However, we can also obtain an Abelian group if we add an inversion operation ${,}^{-1}$ to the monoid. As a result, every element a has an inverse element $a^{-1}$ such that $a\circ a^{-1}=e$ . Furthermore, assume that every element a has its own diagonalization a−1 such that a∘a−1=a−1 and for every inverse element $a^{-1}$ there exists its own diagonalization a such that a−1∘a=a . We define $\bar{e}$ as a∘a−1 . In this way, we obtain an Abelian group with diagonalizations.

The diagonalization $\bar{e}$ , defined as $d(f_{p^{n}}^{t})$ in equation (5.1), is an easy model of reflection for arithmetic functions $f_{p^{n}}^{t}$ . After all, this $\bar{e}$ is not computable for any arithmetic function at time step t . Using e in a calculation means adding new active cells and changing the transition rule in $A_{F}^{O}$ thanks to the oracle o . In extensions of the ZFC model, this manifests itself as an extension M[o] through forcing for the M model.

## 6. Conclusion and discussion

As we have shown in our previous works [12,14], organisms produce more outputs than inputs, which is a strong indication of rudimentary consciousness. This is significant not only because it is not calculable or deterministic, but also owing to the criterion of independence. Let us define a conscious reaction as one where two completely independent stimuli are perceived by the organism as dependent, and this joint perception generates a response. This joint view is considered a manifestation of consciousness. When two completely independent stimuli act on an organism $A^{O}$ , their combined state is perceived together by the oracle O , making them no longer independent, which then generates a joint response. This process can be linked to producing more outcomes than inputs. For this to be possible, the oracle O must add something to the jointly perceived stimuli, thus rendering them interdependent. The crucial point here is that the initial stimuli are fully independent, since if they were initially dependent, we could not determine whether the output conclusions are generated externally or internally by O . Continuing this reasoning, we can mathematically define a self-awareness as a diagonalization a−1 for all the responses a at the time step t . These elements a−1 absorb the responses a , providing maximum closure of all elements into a whole $\bar{e}$ presented as a∘a−1 . Therefore, a composition with such a whole $\bar{e}$ yields this whole itself. This $\bar{e}$ is a form of self-awareness, when the organism $A^{O}$ in its responses to external stimuli may absorb all of these stimuli.

Theoretically, this article is a continuation of our research in the field of designing consciousness as a purely mathematical phenomenon [4,5]. However, now we have looked at mycelium as a concrete example of computation in the framework of oTM, which we previously defined in the language of pure mathematics. Practically, this is a continuation of the study of fungi as automata [8,9,38,39], as well as experimental research [1] according to which a certain orchestration is found in the reactions of the mycelium, so that fungi respond to anaesthesia, demonstrating the presence of a minimal level of consciousness.

Hence, our research remains in certain analogy with the hypothesis of orchestrated objective reduction [40] which suggests that consciousness arises from quantum processes occurring in structures called microtubules within cells. According to this hypothesis, these quantum processes contribute to consciousness by collapsing the quantum state of microtubules into classical states, thereby influencing neural activity and ultimately giving rise to conscious experience. The term ‘orchestrated’ emphasizes the idea that these quantum processes are somehow coordinated or orchestrated in a way that leads to coherent conscious experience, while ‘objective reduction’ refers to the collapse of the quantum state to a classical state. According to Penrose & Hameroff [40], the phenomenon of consciousness is possible owing to chemical microprocesses in an individual cell, such as microtubules and actin filaments [12]. However, we contend that even a single cell exhibits a rudimentary form of consciousness, evident in its capacity to hybridize its behaviour (definition 5). This is demonstrated by its ability to produce more outputs than inputs during the computational process. This model of more outputs than inputs in computation is well formalized by an oTM. Thus, the specificity of our approach is that we consider consciousness not as an event of the collapse of quantum states into classical ones, but as a purely mathematical phenomenon in the appearance of reflection during calculations with the emergence of diagonalization $\bar{e}$ . Quantum processes occurring in the brain can be a source for true mathematically random internal stimuli acting on the level of cells and its constituents which thus enable conscious reactions. Even in the case of comparatively simple organisms like mycelium their behaviour can be well represented as a cellular automaton with an oracle and the mathematical form of consciousness and random reactivity are still applied. This means that the mycelium actually has a minimal form of consciousness in this sense.

The concept of identifying parallels to conscious behaviour in oTMs, as applied in this article, is based on several key observations:

- consciousness embodies a system’s self-awareness and comprehension of its existence within its surrounding environment, which is often unpredictable and random, since stimuli are often independent in advance, but they may be treated as dependent by the system;
- the environment exerts its influence on the system through random independent stimuli, prompting joint responses and reactions;
- these stimuli induce changes in the system, which then integrates and adapts to them;
- over time, the system comprehends and internalizes these changes, transforming the initially random and independent stimuli into familiar elements;
- the influence of stimuli on the system can range in intensity, sometimes to the extent of being disregarded altogether. As a result, stimuli do not always elicit a clear-cut reaction; instead, they may trigger a blend or fusion of responses, leading to hybridized reactions (definition 5);
- reflection regarding stimuli manifests itself in voluntary changes in their potential impact so that stimuli may be even absorbed and neglected; and
- self-reflection or self-awareness is found when cycles appear owing to the maximization of reflection with changes in stimulus intensity, which is expressed through a diagonalization.

In essence, this framework posits that conscious behaviour in oTMs can be analogized to the cognitive processes involved in perceiving, adapting to and interpreting stimuli within a dynamic environment. These complex phenomena can be studied in simple organisms, such as mycelium as a specific computational process within the framework of fungal cellular automata with an oracle.

In the future, we plan to undertake a series of experiments involving fungal mycelium to demonstrate how a cellular automaton with an oracle can effectively simulate the mycelium’s response to external stimuli, while also identifying instances of diagonalization $\bar{e}$ . These experiments are particularly challenging owing to the extended duration required, often exceeding a year. Furthermore, conducting such experiments necessitates a robust mathematical model. We intend to use the framework presented in this article to develop the necessary model and guide our experimental design. The concept of diagonalization $\bar{e}$ is integral to our study. It refers to a specific mathematical property that allows the system to maintain consistency and coherence across different states and inputs. Identifying and understanding these diagonalization manifestations within the mycelium’s behaviour will provide deeper insights into its computational capabilities, demonstrating a form of self-awareness.

## Data Availability

This article has no additional data.

## Footnotes

^1^
Let us be reminded that ${𝐙}^{n}$ means a Cartesian product of 𝐙 , while ${𝐙}_{p}$ is a set of p -adic integers.

^2^
Rigid behaviour refers to behaviour that is deterministic as understood in behaviourism.

^3^
This ability can be mimicked using deep learning.
